# Supplementary figures and images for: A Machine Learning-Based Online Prediction Tool for Predicting Short-Term Postoperative Outcomes Following Spinal Tumor Resections
Source: Cancers (Basel). 2023 Jan 28;15(3):812. doi: 10.3390/cancers15030812 (PMC9913622; doi:10.3390/cancers15030812)

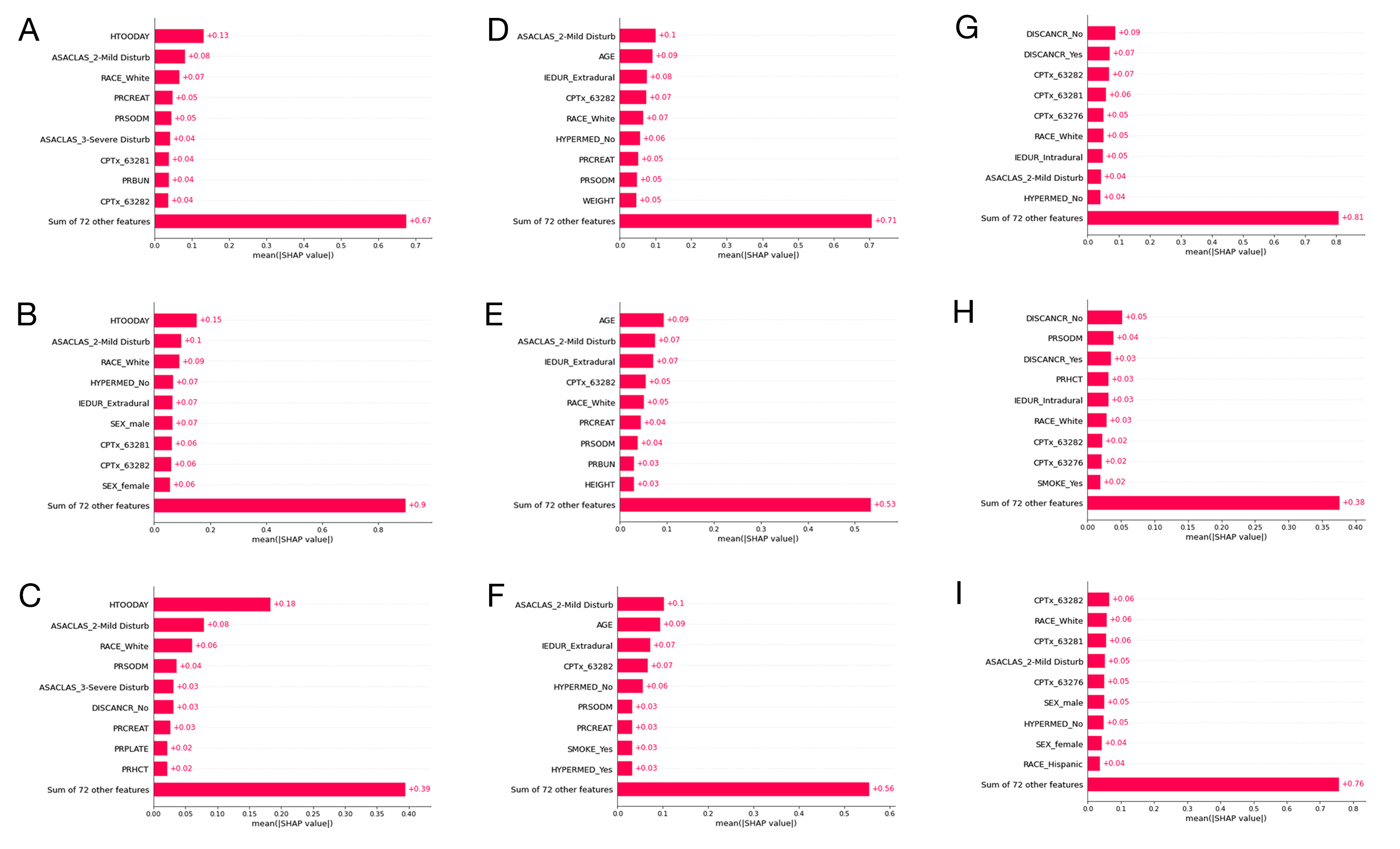

Supplement: Supplementary file 1 [file cancers-15-00812-s001.zip › Supplementary Figure S1.tif]
